# Supplementary material for: Cyclin-Dependent Kinase Inhibitor 2A/B Homozygous Deletion Prediction and Survival Analysis
Source: Brain Sci. 2023 Mar 25;13(4):548. doi: 10.3390/brainsci13040548 (PMC10136851; doi:10.3390/brainsci13040548)
Supplement: Supplementary file 1 [file brainsci-13-00548-s001.zip › Supplementary Table.pdf]

Supplementary Table S1 Feature Selection Results of CDKN2A/B homozygous deletions

	Factor	Coefficients
1	square_gldm_DependenceVariance_edema_ Ga-T1w	0.065179114
2	gradient_firstorder_Kurtosis_tumor_ Ga-T1w	-0.004171261
3	lbp-3D-m1_firstorder_Mean_tumor_ Ga-T1w	-0.017304921
4	square_gldm_LowGrayLevelEmphasis_edema_ Ga-T1w	0.012158874
5	wavelet-HLL_firstorder_Median_tumor_ Ga-T1w	-0.017739141
6	wavelet-LLH_firstorder_Kurtosis_tumor_ Ga-T1w	-0.010943257
7	lbp-3D-m2_firstorder_MeanAbsoluteDeviation_tumor_ Ga-T1w	0.014542148
8	exponential_glszm_LargeAreaHighGrayLevelEmphasis_edema_ Ga-T1w	0.046349516
9	square_firstorder_Minimum_edema_ Ga-T1w	-0.008545986
10	gradient_firstorder_Kurtosis_tumor_Flair	-0.012124982
11	logarithm_glrlm_LongRunHighGrayLevelEmphasis_tumor_Flair	-0.007258985
12	wavelet-LHL_firstorder_Skewness_tumor_Flair	-0.02270516
13	lbp-3D-k_firstorder_Variance_edema_Flair	-0.003945285
14	wavelet-HLH_glszm_ZoneEntropy_edema_Flair	-0.00041629
15	original_firstorder_Minimum_tumor_ Ga-T1w	-0.028830772
16	logarithm_glcm_MCC_edema_ Ga-T1w	-0.006782412
17	original_glrlm_ShortRunLowGrayLevelEmphasis_edema_ Ga-T1w	0.006950688
18	wavelet-LLL_glcm_Correlation_edema_ Ga-T1w	-0.036642363
19	wavelet-LLL_glrlm_LongRunHighGrayLevelEmphasis_tumor_Flair	-0.007561305
20	wavelet-LLL_glszm_SmallAreaLowGrayLevelEmphasis_edema_ Ga-T1w	0.001820441
21	logarithm_firstorder_Kurtosis_edema_ Ga-T1w	0.024500913
22	wavelet-HHL_firstorder_Mean_tumor_ Ga-T1w	0.002108211
23	logarithm_firstorder_Skewness_tumor_Flair	0.001675014
24	gradient_ngtdm_Contrast_tumor_ Ga-T1w	0.048993383
25	gradient_glszm_SmallAreaLowGrayLevelEmphasis_edema_Flair	0.003244593
26	wavelet-LHL_glszm_LargeAreaLowGrayLevelEmphasis_edema_ Ga-T1w	0.014447617
27	squareroot_firstorder_Kurtosis_edema_Flair	-0.01672829
28	exponential_glszm_SmallAreaLowGrayLevelEmphasis_tumor_Flair	-0.006227729
29	wavelet-HHH_gldm_DependenceEntropy_edema_ Ga-T1w	-0.000632322
30	wavelet-LHH_gldm_LargeDependenceLowGrayLevelEmphasis_edema_Flair	0.010099499
31	lbp-3D-k_firstorder_Range_edema_Flair	-0.039733575

32	wavelet-HHL_gldm_LargeDependenceLowGrayLevelEmphasis_edema_ Ga-T1w	0.013258808
33	exponential_firstorder_Range_edema_Flair	0.025264075

Supplementary Table S2 Feature Selection Results of Survival Analysis

序号	feature	Coefficients
1	square_gldm_SmallDependenceLowGrayLevelEmphasis_tumor_Flair	0.016989261
2	square_glszm_SmallAreaLowGrayLevelEmphasis_tumor_ Ga-T1w	0.012057786
3	square_gldm_SmallDependenceLowGrayLevelEmphasis_tumor_ Ga-T1w	0.06312064
4	original_gldm_SmallDependenceLowGrayLevelEmphasis_tumor_Flair	0.040602189
5	square_gldm_SmallDependenceLowGrayLevelEmphasis_edema_Flair	0.047633716
6	wavelet-LLL_glszm_LargeAreaLowGrayLevelEmphasis_edema_ Ga-T1w	0.009459187
7	square_firstorder_Kurtosis_tumor_ Ga-T1w	0.078872822
8	wavelet-HHL_firstorder_Median_tumor_Flair	0.095360369
9	original_gldm_SmallDependenceLowGrayLevelEmphasis_tumor_ Ga-T1w	0.000723061
10	wavelet-LLL_glcm_Correlation_tumor_Flair	-0.042613145
11	exponential_gldm_DependenceVariance_edema_Flair	0.036076706
12	logarithm_firstorder_Kurtosis_edema_ Ga-T1w	0.043348259
13	wavelet-LLL_glszm_LargeAreaHighGrayLevelEmphasis_edema_ Ga-T1w	0.044249824
14	wavelet-LHL_firstorder_Kurtosis_edema_Flair	-0.009457604
15	wavelet-LLL_firstorder_Kurtosis_edema_ Ga-T1w	0.044640465
16	wavelet-HHL_gldm_DependenceEntropy_tumor_Flair	-0.01025543
17	exponential_glszm_SmallAreaEmphasis_edema_Flair	0.009063602
18	exponential_glszm_SmallAreaLowGrayLevelEmphasis_tumor_ Ga-T1w	0.012845736
19	original_glcm_InverseVariance_tumor_Flair	0.053022204
20	wavelet-LHH_firstorder_Median_tumor_Flair	0.051758905
21	wavelet-HLL_glcm_Imc1_edema_Flair	-0.02505621
22	gradient_glszm_SmallAreaLowGrayLevelEmphasis_edema_Flair	0.010234069
23	wavelet-HLL_glcm_ClusterShade_tumor_Flair	-0.003420751

Supplementary Table S3 The results of clinical characteristics

Factor	p-value	HR (95% CI for HR)
CDKN2A/B	0.0025	2.6 (1.4-5)
WHO grade	0.17	3.5(0-Inf)
Sex	0.38	1.5 (0.58-4.1)
Age	0.31	1 (0.98-1.1)
1p/19q	0.47	0.7 (0.26-1.9)
Histologic diagnosis	1.2	-0.64(0.2-1.7)

Supplementary Table S4 The results of survival analysis

Model	Dataset	C-index	SE
Clinial	train	0.624	0.099
Clinial	validation	0.89	0.082
Ga-T1w	train	0.833	0.059
Ga-T1w	validation	0.744	0.151
Flair	train	0.862	0.545
Flair	validation	0.791	0.089
Ga-T1w+Flair	train	0.876	0.046
Ga-T1w+Flair	validation	0.767	0.09
Clinical+MRI	train	0.876	0.044
Clinical+MRI	validation	0.86	0.099
